# Supplementary material for: Integrating Continuous Transepithelial Flux Measurements into an Ussing Chamber Set-Up
Source: Int J Mol Sci. 2024 Feb 13;25(4):2252. doi: 10.3390/ijms25042252 (PMC10889482; doi:10.3390/ijms25042252)
Supplement: Supplementary file 1 [file ijms-25-02252-s001.zip › ijms-2828273-supplementary.pdf]

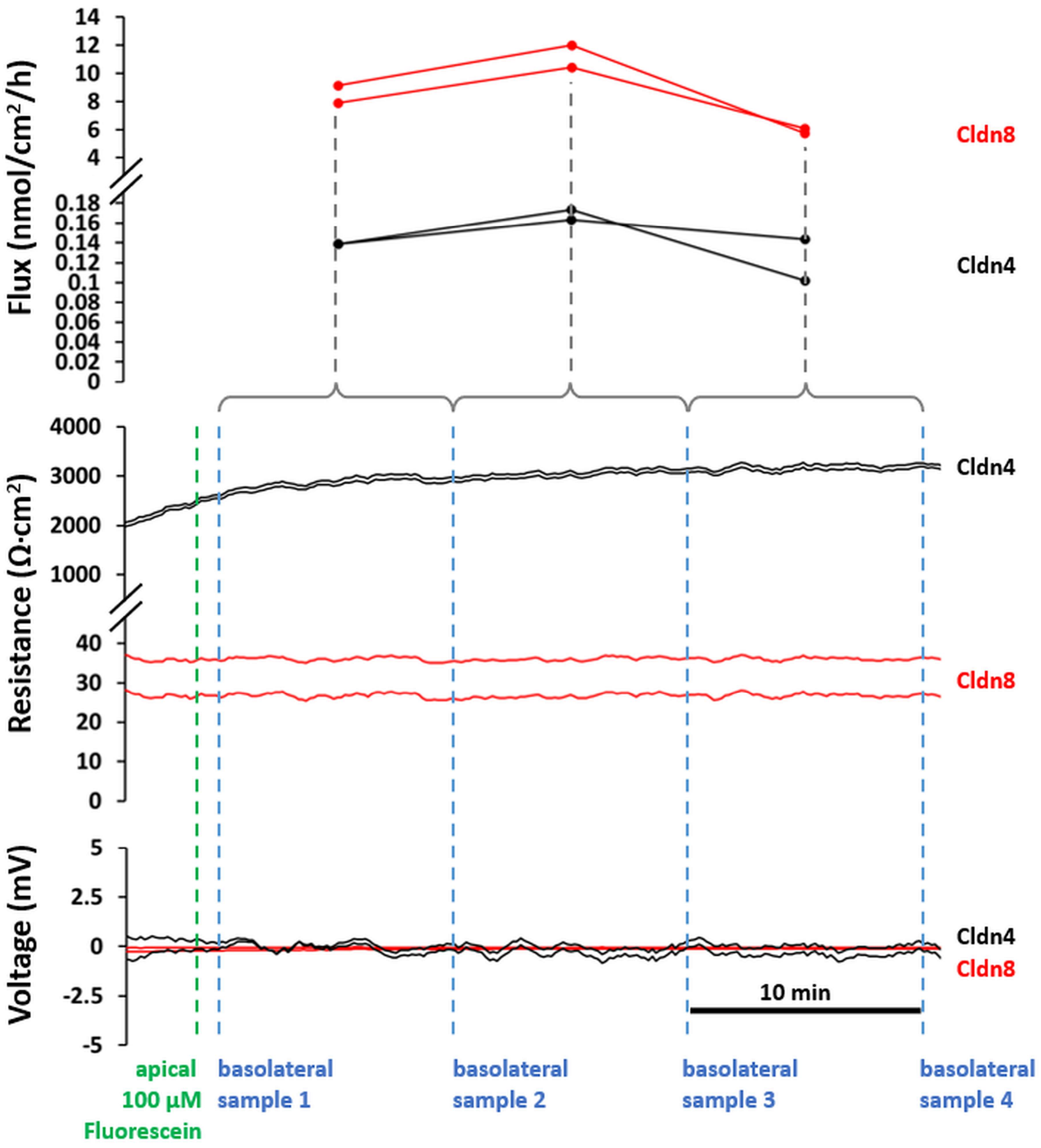

Figure S1: Example of a conventional fluorescein flux experiment. An Ussing chamber experiment was performed on filter supports with Cldn4- and Cldn8-transfected claudin quin KO cell layers, respectively. Transepithelial voltage (bottom traces) was close to zero for all filter supports throughout the experiment. Transepithelial resistance (middle traces) differed greatly (by two orders of magnitude) between Cldn4- and Cldn8-transfected cell layers. 100 µM fluorescein was was added to the apical side (donor compartment) as indicated by the green line. Samples were collected from the basolateral side (acceptor compartment) at four different time points, as indicated by the blue lines, and the fluorescein concentration determined in a plate reader. From these values fluxes were calculated for the three 10 min time intervals between the four readings (upper traces). Fluxes were about two orders of magnitude higher in Cldn8- compared to Cldn4-transfected cell layers.

Table S1: Baseline TER, fluorescein flux and fluorescein permeability values of the employed cell lines.

	**Claudin quin KO + Cldn2**	**Claudin quin KO + Cldn4**	**Claudin quin KO + Cldn8**	**HT-29/B6****
**TER** ($\Omega \cdot cm^2$)	29.7 ± 2.5 n=6	2685 ± 112 n=10	19.1 ± 2.3 n=10	615.5 ± 9.4 n=8
**Fluorescein Flux*** ($nmol/cm^2/h$)	0.63 ± 0.07 n=6	0.14 ± 0.01 n=10	8.15 ± 0.22 n=10	0.10 ± 0.01 n=3
**Permeability** (10^{-6} cm/s)	1.74 ± 0.48 n=6	0.38 ± 0.03 n=10	22.64 ± 0.62 n=10	0.28 ± 0.03 n=3

* Flux values obtained from conventional concentration measurements (utilizing a plate reader) as shown in Figure S1.

Values from Krug S. M., Fromm M., Günzel D. Two-path impedance spectroscopy for measuring paracellular and transcellular epithelial resistance. *Biophys J* **2009, *97*(8), 2202-11
